# The Impact of COVID-19 Vaccination and Infection on the Exacerbation of Myasthenia Gravis

**DOI:** 10.3390/vaccines12111221

**Published:** 2024-10-27

**Authors:** Yuting Jiang, Jingsi Wang, Shengyao Su, Shu Zhang, Qi Wen, Yaye Wang, Ling Li, Jianxin Han, Nairong Xie, Haoran Liu, Yanan Sun, Yan Lu, Li Di, Min Wang, Min Xu, Hai Chen, Suobin Wang, Xinmei Wen, Wenjia Zhu, Yuwei Da

**Affiliations:** 1 Department of Neurology, Xuanwu Hospital, Capital Medical University, Beijing 100053, China; jiang86830929@163.com (Y.J.); wangjs9502@163.com (J.W.); ssy6917@163.com (S.S.); 18366111865@139.com (S.Z.); 13303475918@163.com (Q.W.); 18332058288@163.com (Y.W.); kingsmart0731@126.com (L.L.); hanjianxin2514@sina.com (J.H.); xienairong005233@163.com (N.X.); lhr990116@163.com (H.L.); sunywest@sina.com (Y.S.); dr_stone@126.com (Y.L.); super_dili@163.com (L.D.); wangminxw@126.com (M.W.); doctor_xumin@126.com (M.X.); chenhai@xwhosp.org (H.C.); sbwang1964@163.com (S.W.); xinmei.wen@outlook.com (X.W.); zhwjia@163.com (W.Z.); 2 Department of Neurology, Tianjin 4th Centre Hospital, Tianjin 300140, China; 3 Department of Neurology, Liangxiang Hospital of Beijing Fangshan District, Beijing 102401, China; 4 Department of Neurology, Dalian Municipal Friendship Hospital, Dalian 116001, China

**Keywords:** myasthenia gravis, COVID-19 infection, vaccination, exacerbation, outcomes, omicron variants

## Abstract

Objectives: Myasthenia Gravis (MG) is an autoimmune disorder that can exacerbate for various reasons, including vaccination and infection. This study aimed to investigate the safety of COVID-19 vaccines for MG patients, factors influencing MG exacerbation after COVID-19 infection (MECI), the course and prognosis of MECI, and the impact of COVID-19 vaccine on infected MG patients. Methods: Patients were enrolled from the MG database in the Department of Neurology, Xuanwu Hospital, Capital Medical University. Two questionnaires were administered to collect data concerning COVID-19 vaccination (questionnaire 1, Q1) and infection (questionnaire 2, Q2) during two distinct periods. MG exacerbation was defined as an increase of at least two points in the MG activity of daily living (MG-ADL) score. COVID-19 severity was categorized as “hospitalization” or “home management”; Results: During the first data-collecting period, our database registered 1013 adult patients: 273 (26.9%) had received COVID-19 vaccinations and completed Q1, and 8 (2.9%) experienced MG exacerbation after vaccination. During the second data-collecting period, among the newly registered patients, 366 patients completed Q2. Of these, 244 were infected, with 39 (16.0%) experiencing MECI and 21 (8.6%) requiring hospitalization. Multivariate analysis showed that generalized myasthenia gravis was associated with MECI (OR 3.354, 95% CI: 1.423–7.908, *p* = 0.006). Among the 244 infected patients, 143 had received COVID-19 vaccinations, including 14 who received their booster dose within 6 months before COVID-19 and 129 who were vaccinated more than 6 months before COVID-19. The remaining 101 were unvaccinated. No significant associations were found between COVID-19 vaccination and COVID-19 severity (*p* = 0.292) or MECI incidence (*p* = 0.478); Conclusions: COVID-19 vaccines were found to be safe for MG patients in stable condition. Patients with gMG were more susceptible to experiencing MECI. No significant impact of the vaccine on COVID-19 severity or MECI incidence was observed.

## 1. Introduction

Myasthenia gravis (MG) is an autoimmune disorder primarily affecting the neuromuscular junction, characterized by fluctuating muscle weakness and fatigue [1]. Acute infections are the most frequent triggers for MG exacerbations, accounting for 30% of hospital visits, with vaccinations and antibiotics like aminoglycosides, macrolides, and quinolones posing additional risks [2]. Consequently, in the context of the coronavirus disease 2019 (COVID-19) pandemic caused by severe acute respiratory syndrome coronavirus 2 (SARS-CoV-2), MG management is crucial. Although the global pandemic has been declared over, the virus continues to evolve; the highly mutated Omicron variants became more prevalent in late 2023 and early 2024 [3,4]. Data from the Chinese Center for Disease Control and Prevention reveal that the national positivity rate for SARS-CoV-2 during May and June 2024 ranged from 5.4% to 7.2%, comparable to the influenza virus rate. This rate reached 21.1% in August, indicating a fluctuating prevalence of COVID-19. https://www.chinacdc.cn/jkzt/crb/zl/szkb_11803/ (accessed on 20 September 2024). Therefore, the impact of COVID-19 on MG patients is expected to persist in the future.

As the most effective tool for protecting against infection, the COVID-19 vaccine has been globally promoted and used since the end of 2020. Vaccination against COVID-19 not only reduces hospitalization and mortality rates among MG patients infected with SARS-CoV-2 [5,6], but also offers protection against the exacerbation of MG symptoms following infection [7]. Although current studies have demonstrated the safety of COVID-19 vaccines in MG patients [8], there is still a reserved attitude towards the COVID-19 vaccination among MG patients. A Chinese study indicated that the COVID-19 vaccination rate among MG patients was 25.7%, substantially lower than China’s overall rate of 89.2% [9], suggesting that there is still considerable potential for vaccination among MG patients and the safety of COVID-19 vaccination in MG patients still needs to be proven.

As the initial wave of COVID-19 infections recedes, there is evidence that the memory T-cells developed in response to SARS-CoV-2 do not persist in the long term [10], which could elevate the risk of subsequent infections. With the prospect of MG patients living alongside COVID-19, an investigation into the factors influencing MG’s exacerbation after COVID-19 infection (MECI) is warranted. Nevertheless, studies have emphasized the course of COVID-19 in MG patients, but few of them have specifically concentrated on the deterioration of MG symptoms following COVID-19 infection. Furthermore, most of the existing research was conducted during the early phases of the pandemic, driven by the SARS-CoV-2 Alpha and Delta variants. This differs from the situation with the Omicron subvariants, which led to a concentrated outbreak in China at the end of 2022, characterized by a milder disease but with a higher transmission rate [11]. However, there is no study describing the features of MECI with the Omicron variants.

In this study, we conducted an analysis of the safety of COVID-19 vaccines in MG patients and the factors influencing MECI. We focused on the assessment of the course and prognosis of MECI. We also explored the effects of the COVID-19 vaccine on the outcome of MG patients infected with COVID-19, including the severity of COVID-19 and the incidence of MECI.

## 2. Methods

### 2.1. Study Design

In response to the evolving stages of the COVID-19 epidemic in China, we conducted two surveys and collected data through phone calls or face-to-face interviews with distinct groups of patients during two different periods. The first period was from December 2020 to November 2021, focusing on COVID-19 vaccination. The second period was from December 2022 to March 2023, following a concentrated outbreak, and focused on COVID-19 infection.

### 2.2. COVID-19 Vaccination and COVID-19 Infection

Data collection was based on a prospective MG database in the Department of Neurology, Xuanwu Hospital, Capital Medical University, Beijing, China. All patients in the database met the diagnostic criteria for MG and had provided written informed consent. The Ethics Committee of Xuanwu Hospital approved the study ([2017]084 and [2022]082). Demographic and MG clinical data were collected from the MG database, including age, gender, MG subtype (ocular myasthenia gravis or generalized myasthenia gravis, oMG or gMG), the detection of antibodies against the acetylcholine receptor (AChR), thymoma history, disease duration, MG stability duration, and MG baseline treatment (pyridostigmine, prednisolone, and immunosuppressive drugs).

MG activity of daily living (MG-ADL) scores were collected before and after COVID-19 vaccination and infection. MG exacerbation was defined as an increase of at least two points in the MG-ADL score within 6 weeks post-vaccination or post-infection, following a period of at least one month of stable MG, excluding other factors that could contribute to MG exacerbation.

In order to evaluate the safety of COVID-19 vaccination among MG patients, we surveyed 1013 adult patients, who constituted the complete set of MG adult patients in our database by the end of the first data collection period. We included those who had received COVID-19 vaccinations and excluded those with a history of SARS-CoV-2 infection. The participants were categorized into two groups: those who experienced MG exacerbation after COVID-19 vaccination (MECV) and those who did not (NMECV). The severity of MG at the time of vaccination was evaluated by MG-ADL score and classified as stable (0 points, 0 p), mild (1–2 points, 1–2 p), moderate (3–5 points, 3–5 p), and severe (6 points or more, ≥6 p). Participants were asked about their history of COVID-19 vaccination, including vaccination status, the vaccine type, number of doses received, and the dates of vaccination. Full vaccination was defined as the administration of a single dose of adenovirus vector vaccine, two doses of inactivated or mRNA vaccines, or three doses of a recombinant vaccine. To identify the factors influencing MECI, we conducted a survey among a subset of patients in our database who were predominantly newly registered during the second data collection period. Adult patients infected with SARS-CoV-2 were enrolled, excluding those whose disease onset occurred after the infection. We also categorized participants into two groups: those who experienced MECI and those who did not (NMECI). Infection was confirmed through laboratory testing, including polymerase chain reaction (PCR) or antigen detection from nasopharyngeal swabs. The follow-up duration for patients with MG exacerbation was based on the routine follow-up time points established in our database.

To characterize the clinical features of MECI, we documented the clinical manifestations, subsequent treatment adjustments, and patient outcomes, as well as the time of onset of MECI after COVID-19. To evaluate the impact of COVID-19 vaccines on the outcomes of infected MG patients, vaccination details were collected, consistent with the first questionnaire. Booster dose was defined as an extra vaccine administered after full vaccination. Given the waning efficacy of COVID-19 vaccines over time, we divided patients into three groups according to their vaccination status: Group 1 included those who received their booster dose within 6 months before COVID-19, group 2 comprised patients who were vaccinated more than 6 months before COVID-19, and group 3 consisted of 101 unvaccinated patients. This comparative approach is supported by Zeng et al.’s research [12], which demonstrates that neutralizing antibodies remain detectable 6 months after a CoronaVac booster dose, but decline below detectable levels by 6 months after the second dose. The outcome indicators were the severity of COVID-19 and the incidence of MECI. The severity of COVID-19 was categorized based on hospitalization status as “hospitalization” or “home management”. Clinical manifestations of COVID-19 were also collected.

### 2.3. Statistical Analyses

Statistical analyses were conducted using IBM SPSS version 26.0 (IBM Corp., Armonk, NY, USA). Quantitative data were analyzed using the non-parametric Mann–Whitney U test, while categorical data were analyzed using the chi-square test or Fisher’s exact test. The results for categorical variables were presented as frequencies (percentages), and results for continuous variables as median (range) or median (Q1, Q3), where Q1 and Q3 represent the first and third quartiles, respectively. In addition, multivariable logistic regression analysis was performed to investigate the factors influencing MECI. We employed the Bonferroni method for comparisons across multiple groups. Two-sided *p* values of less than 0.05 were considered statistically significant.

## 3. Results

### 3.1. COVID-19 Vaccine Safety in MG Patients

#### 3.1.1. Baseline Characteristics of COVID-19 Vaccine Recipients

**Figure 1:**
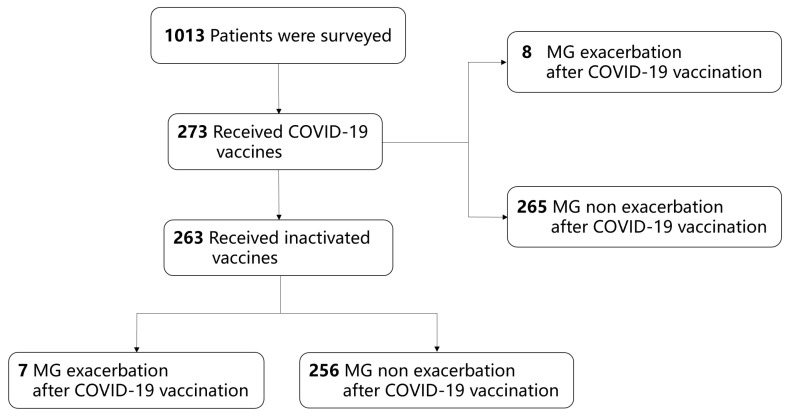
Patient enrollment process in COVID-19 vaccination survey.

During the first data-collecting period, our database registered 1013 adult patients: 273 (26.9%) received COVID-19 vaccinations and enrolled and 8 of them experienced MECV. Inactivated vaccines were administered in 263 (96.3%) patients (CoronaVac of 179, BBIBP-CorV of 84) and 7 of them experienced MECV. The remaining 10 patients were administered recombinant (ZF2001), adenovirus vector (Ad5-nCoV), or mRNA (BNT162b2) vaccines. A total of 256 patients (93.8%) finished vaccination. During the data collection period, none of the participants had been infected with SARS-CoV-2. The patient enrollment process is shown in Figure 1.

There were 242 (88.6%) patients with stable or mild diseases (MG-ADL 0p–2p), and 31 (11.4%) with moderate to severe diseases (MG-ADL 3p–9p) at the point of vaccination. Immunosuppressants were used by 109 (39.9%) patients. Among them, 93 (85.3%) patients were treated with monotherapy, including steroids (*n* = 52), tacrolimus (*n* = 36), azathioprine (*n* = 2), methotrexate (*n* = 2), and mycophenolate mofetil (*n* = 1). The rest (14.7%) were using steroids in combination with one nonsteroidal immunosuppressant. Other patients were taking pyridostigmine alone (21.6%) or discontinued medications due to clinical remission (38.5%) (Table 1).

#### 3.1.2. Factors Influencing MECV

Among 273 patients, 8 (2.9%) experienced MECV, while 265 (97.1%) did not. No significant differences were found between MECV and NMECV groups in terms of gender, age, oMG or gMG, antibodies against AChR, thymoma, disease duration, MG severity, treatment at the time of vaccination, vaccine type, or full vaccination status (Table 1). Since the majority (263/273, 96.3%) of the participants received inactivated vaccines, we also analyzed the factors influencing MECV in these patients. The exacerbation was not associated with the above variables either (Supplementary Table S1).

#### 3.1.3. Course and Prognosis of Patients with MECV

Eight patients experienced MECV (Supplementary Table S2): seven received the inactivated vaccine, five (62.5%) exhibited exacerbation after the second dose, and most (6/8, 75.0%) experienced MECV within about 2 weeks. The median time to MG deterioration was 12 days (range 2–30). Extraocular muscles were most affected, with no respiratory involvement. Of eight patients, seven (87.5%) were asymptomatic at baseline. The MG-ADL scores increased by a median of four points (range 2–12). Medication regimens were adjusted for seven patients, including the addition or escalation of immunosuppressants for four. By final follow-up, six achieved Minimal Manifestation Status (MMS), defined as the absence of symptoms or functional limitations from MG, within a median of 30 days (range 15–150 days), while one who declined immunotherapy worsened.

### 3.2. COVID-19 Infection in MG Patients

#### 3.2.1. Baseline Characteristics of Patients Infected with COVID-19

**Figure 2:**
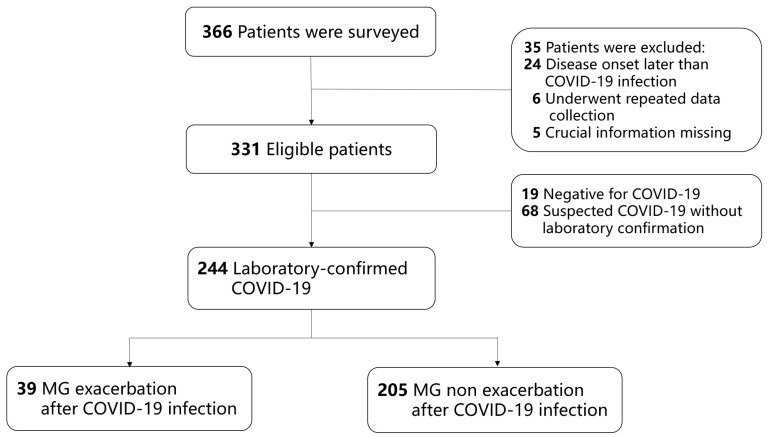
Patient enrollment process in COVID-19 infection survey.

During the second data-collecting period, among the newly registered patients, 366 patients were surveyed. A total of 244 patients infected with COVID-19 were enrolled, comprising 202 with positive PCR tests and 42 with positive antigen tests. Of the 244 patients, 39 (16.0%) experienced MECI, whereas 205 (84.0%) did not (NMECI). A total of 143 (58.6%) patients received COVID-19 vaccinations, and 134 of them received inactivated vaccines. The patient enrollment process is shown in Figure 2.

The MECI group had a notably higher proportion of gMG patients (31/39, 79.5%) compared to the NMECI group (109/205, 53.2%, *p* = 0.002), suggesting that gMG could be a contributing risk factor for MECI. We found no difference between MECI and NMECI groups in terms of the other collected data, including gender, age, antibodies against AChR, thymoma, disease duration, treatment at infection, vaccination status, COVID-19 severity, and COVID-19’s clinical manifestations (Table 2). Multivariable logistic regression analysis proved that gMG (OR 3.354, 95% CI: 1.423–7.908, *p* = 0.006) was an independent risk factor of MECI (Table 3).

#### 3.2.2. Course and Prognosis of Patients with MECI

Table 4 shows the clinical course and prognosis for 39 patients with MECI in detail. The median onset of MECI was 3 days (range 1–15). The median MG-ADL score at baseline was 0 (range 0–7), which increased to 3 (range 2–13) after infection, with a median change in score of 3 (range 2–8).

The most prevalent symptoms of MECI were ptosis and limb weakness, with each affecting around half of the patients. Respiratory symptoms were also common, occurring in 41.0% of the total cases, while bulbar muscle weakness was observed in 23.1% of patients. More than half of the gMG patients (16/31, 51.6%) reported varying degrees of respiratory symptoms, with five requiring hospitalization, accounting for 83.3% of all hospitalizations among MECI patients. Among the patients with MECI, none experienced myasthenic crisis.

Out of the twelve patients whose medication regimens were modified following infection, nine required the addition of new medications, including IVIG and steroids (*n* = 3), steroids alone (*n* = 2), tacrolimus (*n* = 1), corticosteroid–mycophenolate combination (*n* = 1), and acetylcholinesterase inhibitors (*n* = 2). The other three patients escalated their medications, with two boosting their steroid doses and one their tacrolimus dose.

Within 3 months after COVID-19 infection, 23 patients (59.0%) experienced an improvement in their symptoms. Among the 12 patients who modified medication regimens, 8 (66.7%) showed an MG improvement (median improvement time abbreviated as MIT: 10 days; range 3–30), with 3 (25.0%) returning to MMS, and no patients continued to deteriorate. Among the 27 patients who maintained their original treatment plans, 15 (55.6%) improved (MIT: 13 days, range 7–40), with 3 (11.1%) returning to MMS, and 2 worsened.

### 3.3. The Impact of COVID-19 Vaccine on Infected MG Patients

#### COVID-19 Vaccine Effects on MG Patients Infected with COVID-19

Among the 143 vaccinated patients, the median interval from the last vaccine dose to infection was 13 months. We compared three groups based on their vaccination status. The first group (vaccination 1 group) included 14 patients who received their booster dose within 6 months before COVID-19, with all having received inactivated vaccines. The second group (vaccination 2 group) comprised 129 patients who were vaccinated more than 6 months before COVID-19. The third group (unvaccination group) consisted of 101 unvaccinated individuals (Table 5).

There was an imbalance in the MG duration and utilization of immunosuppressive therapy among the three groups. However, we found no significant impact of vaccination on the severity of COVID-19 (*p* = 0.292) and the incidence of MECI (*p* = 0.478).

## 4. Discussion

We found that the incidence of MECV was low (8/273, 2.9%). gMG might be a contributing risk factor for MECI. We did not find significant effects of the COVID-19 vaccine on the severity of COVID-19 and the incidence of MECI.

In studies focusing on the safety of COVID-19 vaccination for MG patients, the reported rates of MECV vary. An Italian study reported a 7.7% (8/104) exacerbation rate [13], Sansone and Bonifati et al. reported 5% (4/80) [14], a Japanese study noted 1% (3/294) [15], and Doron et al. observed an 8.7% (13/150) exacerbation rate [16]. Our study revealed an incidence rate of 2.9% (8/273), lower than most of the previously reported data. This discrepancy could potentially be attributed to the criteria for MG exacerbation used in our study, requiring at least one month of stable MG conditions before vaccination. And our finding was in line with Gamez et al.’s study, which reported a 2.2% exacerbation rate among patients on stable therapy for at least 180 days before vaccination [17]. MECV was found to be associated with exacerbation in the past six months [18].

In our study, MG exacerbation typically occurred within 2 weeks after vaccination, consistent with other research findings. Notably, 62.5% (5/8) of patients experienced MG exacerbation following the second dose, aligning with the results reported by Sansone and Bonifati et al. [14]. The underlying cause may be the more robust immune response induced by the second dose [19]. The median increase in MG-ADL scores of MECV was four points; four cases (50%) required the addition or escalation of immunosuppressants, and 50% of patients with MECV achieved remission within one month. This differs from the findings reported by Ruan et al. [9], where the average increase in MG-ADL was 2.2, and MG exacerbation typically resolved without intervention in 1–2 weeks [9,13,20]. This discrepancy may be attributed to the lower proportion of patients in our cohort (39.9%) who received immunosuppressive therapy due to well-controlled MG, which was significantly lower than the rates observed in other studies (49.1%, 73.8%, and 80.7%), potentially resulting in a higher degree of MG exacerbation.

After COVID-19 infection, 16% (39/244) of patients in our study experienced exacerbation, which is consistent with the previously reported rates of 11.6% to 19.3% [21,22,23,24]. We observed that gMG patients were more susceptible to MECI, possibly due to the activation of the immune response in this group. Research suggests that the dysfunction of and reduction in regulatory T (Treg) cells, which play a crucial role in negatively regulating immune responses, may contribute to the exacerbation of MG conditions [25]. Compared to patients with oMG, gMG patients have lower numbers of Treg cells [26]. During COVID-19 infection, the increase in inflammatory cytokines such as IL-2 can downregulate Treg cells [27], potentially further contributing to the activation of the immune response in gMG patients.

Concerning the profile of MG exacerbation, our research indicated that the median increase in MG-ADL scores for MECI was 3 points, similar to the 4.8 points reported in Peric et al.’s study [21]. Our study showed 41.0% of patients had respiratory muscle involvement, which may significantly increase the risk of myasthenic crisis, a potentially life-threatening condition [28]. In our study, three patients received a combination therapy of corticosteroids and IVIG due to MECI, with no deaths attributed to worsening MG symptoms. Other research indicated that 2.8% (3/108) of COVID-19-infected MG patients were admitted to the intensive care unit, and two of them died [24]. The prognosis of MECI was observed within 3 months, with 59% showing improvements, while 35.9% remained unchanged and 5.1% worsened. Patients who adjusted their medication showed a higher rate of improvement compared to those who did not, and none of them experienced continued MG deterioration. To our knowledge, this is the first study focusing on the course and prognosis of MG exacerbation following Omicron infection.

Regarding the effects of the COVID-19 vaccine on MG patients infected with COVID-19, a recent study by Li observed no impact on the change in MG-ADL score after COVID-19 infection and the severity of COVID-19 [24]. Additionally, a survey revealed that a lack of COVID-19 vaccination was a risk factor for MECI, and the rate of hospitalization was higher than those who were vaccinated [7,29]. However, neither of the research considered the interval between vaccination and infection. Considering that the efficacy of COVID-19 vaccine declines over time, and a study demonstrated that the CoronaVac booster can keep neutralizing antibody levels detectable for a period of 6 months [12], we compared three groups of patients based on their vaccination status to assess the impact of the COVID-19 vaccination on the outcome of infected MG patients, but found no significant correlation.

A possible reason for this might be that the divergent mutations of Omicron variants increase its immune evasion, which may result in inadequate vaccine protection. Research indicated that the Omicron form may evade immunization twice as often as the Delta version [30]. According to Cao et al., most individuals kept the neutralizing titers against ancestral Wuhan-Hu-1 but lost the neutralizing activities against Omicron subvariants at 10–12 months post the booster dose of CoronaVac [31]. Additionally, it is important to take into account that there was a higher proportion of immunosuppressive therapy use in the unvaccinated group at baseline. The impact of immunosuppressive therapy on COVID-19 remains uncertain. An early German study identified this to be a risk factor for a severe disease course of COVID-19 [32], but other studies reported no impact of immunosuppressants on COVID-19 [7,33,34]. Furthermore, research has indicated that the immunosuppressants were associated with an elevated MG-ADL score after COVID-19 [24]. A more detailed subgroup analysis should be conducted.

Our study is the first to characterize the features of MECI that are associated with the Omicron variants and to assess the effect of COVID-19 vaccinations in MG patients infected with COVID-19, taking into account the vaccine’s diminishing efficacy over time. However, several limitations should be considered: (1) The potential for recall bias in the retrospective study could result in the underreporting of mild MG exacerbations. (2) The extended time interval between COVID-19 vaccination and infection in our cohort raises questions about the persistence of vaccine-induced immunity, and our study did not include COVID-19-neutralizing antibody testing to assess the vaccine’s effectiveness at the time of infection. (3) The analysis of the impact of vaccination on the outcome of infected MG patients is limited by the small sample size and the imbalance in baseline covariates, which may lead to a reduction in statistical power. (4) Due to the absence of extended follow-up data for all patients, we were unable to perform a sensitivity analysis, potentially limiting the generalizability of our findings regarding long-term disease progression in MG patients post vaccination or infection. Consequently, further research is necessary to explore and establish an effective long-term COVID-19 vaccination strategy for MG patients.

## 5. Conclusions

In conclusion, COVID-19 vaccines were confirmed to be safe for MG patients in a stable condition. Patients with gMG were more likely to experience MECI. We did not find a significant impact of COVID-19 vaccination on COVID-19 severity or MECI incidence.

## Figures and Tables

**Table 1:** Clinical characteristics of MG patients receiving COVID-19 vaccines.

	Total (*n* = 273)	MECV (*n* = 8)	NMECV (*n* = 265)	*p*
Gender				1.000
Female	113 (41.4)	3 (37.5)	110 (41.5)	
Male	160 (58.6)	5 (62.5)	155 (58.5)	
Age, median (Q1, Q3), y	56 (41, 64)	53 (41, 70)	56 (41, 64)	0.856
MG Type				0.789
gMG	107 (39.2)	4 (50.0)	103 (38.9)	
oMG	166 (60.8)	4 (50.0)	162 (61.1)	
AChR-Ab	205 (75.1)	7 (87.5)	198 (74.7)	0.683
Thymoma	22 (8.1)	2 (25.0)	20 (7.5)	0.129
MG duration, median (Q1, Q3), m	37 (26, 57)	47 (32, 70)	37 (26, 56)	0.399
MG-ADL scale at vaccination				0.598
0 (stable)	211 (77.3)	7 (87.5)	204 (77.0)	
1–2 (mild)	31 (11.4)	0	31 (11.7)	
3–5 (moderate)	25 (9.2)	1 (12.5)	24 (9.1)	
≥6 (severe)	6 (2.2)	0	6 (2.3)	
MG-ADL score at vaccination, median (range)	0 (0-9)	0 (0-3)	0 (0-9)	0.517
MG stabilization time before vaccination, median (Q1, Q3), m	12 (5, 24)	24 (4, 48)	12 (5, 23)	0.283
Treatment at vaccination				0.660
No medication	105 (38.5)	4 (50.0)	101 (38.1)	
Pyridostigmine alone	59 (21.6)	2 (25.0)	57 (21.5)	
IST *	109 (39.9)	2 (25.0)	107 (40.4)	
Vaccine type				0.261
Inactivated vaccine	263 (96.3)	7 (87.5)	256 (96.6)	
Recombinant protein vaccine	8 (2.9)	1 (12.5)	7 (2.6)	
Adenovirus vector vaccine	1 (0.4)	0	1 (0.4)	
mRNA vaccine	1 (0.4)	0	1 (0.4)	
Full vacciantion				0.082
Yes	256 (93.8)	6 (75.0)	250 (94.3)	
No	17 (6.2)	2 (25.0)	15 (5.7)	

Abbreviations: MG, myasthenia gravis; MECV, MG exacerbation after COVID-19 vaccination; NMECV: no MG exacerbation after COVID-19 vaccination; y, years; oMG, ocular MG; gMG, general MG; AChR-Ab, acetylcholine receptor antibody; Q1, the first quartile; Q3, the third quartile; m, months; MG-ADL, myasthenia gravis activity of daily living; IST, immunosuppressants.* IST: including steroids and non-steroid immunosuppressants.

**Table 2:** Factors influencing MECI.

	Total (*n* = 244)	MECI (*n* = 39)	NMECI (*n* = 205)	*p*
Gender				
Female	108 (44.3)	18 (46.2)	90 (43.9)	0.795
Male	136 (55.7)	21 (53.8)	115 (56.1)	
Age, median (Q1, Q3), y	58 (43, 68)	59 (43, 69)	58 (43, 68)	0.887
MG type				
gMG	140 (57.4)	31 (79.5)	109 (53.2)	0.002
oMG	104 (42.6)	8 (20.5)	96 (46.8)	
AChR-Ab	196 (80.3)	30 (76.9)	166 (81.0)	0.560
Thymoma	38 (15.6)	7 (17.9)	31 (15.1)	0.655
MG duration (Q1, Q3), m	38 (18, 71)	47 (15, 82)	38 (20, 68)	0.538
Treatment at infection				
No IST	96 (39.3)	16 (41.0)	80 (39.0)	0.307
Steroids only	60 (24.6)	6 (15.4)	54 (26.3)	
Non-steroids IST	88 (36.1)	17 (43.6)	71 (34.6)	
Vaccination status				
Vaccinated	143 (58.6)	25 (64.1)	118 (57.6)	0.447
Unvaccinated	101 (41.4)	14 (35.9)	87 (42.4)	
COVID-19 severity				
Home management	223 (91.4)	33 (84.6)	190 (92.7)	0.182
Hospitalization	21 (8.6)	6 (15.4)	15 (7.3)	
COVID-19 Clinical Manifestations				
Fever	194 (79.5)	34 (87.2)	160 (78.0)	0.195
Sore throat	51 (20.9)	8 (20.5)	43 (21.0)	0.948
Cough	143 (58.6)	21 (53.8)	122 (59.5)	0.510
Expectoration	46 (18.6)	8 (20.5)	38 (18.5)	0.772
Muscle aches	71 (29.1)	9 (23.1)	62 (30.2)	0.366

Abbreviations: MECI, MG exacerbation after COVID-19 infection; NMECI: no MG exacerbation after COVID-19 infection.

**Table 3:** Multivariable logistic regression analysis of factors for MECI.

	OR Estimate	OR (95% CI)	*p*
Male	0.996	0.481–2.062	0.992
Age	1.000	0.977–1.025	0.976
Generalized MG	3.354	1.423–7.908	0.006
Hospitalization	2.256	0.771–6.601	0.137
IST	1.049	0.504–2.183	0.899

Abbreviations: OR, odds ratio; CI, confidence interval.

**Table 4:** Course and prognosis of patients with MECI.

	MECI (*n* = 39)	gMG-MECI (*n* = 31)	oMG-MECI (*n* = 8)
COVID-19 to MG worsening time, median (range), d	3 (1, 15)	3 (1, 15)	2.5 (1, 5)
MG-ADL, median (range)			
Pre-Infection MG-ADL score	0 (0, 7)	0 (0, 7)	0 (0, 4)
Post-Infection MG-ADL score	3 (2, 13)	3 (2, 13)	3 (2, 6)
Score change	3 (2, 8)	3 (2, 8)	3 (2, 4)
MG exacerbation manifestation			
Ptosis	20 (51.3)	14 (45.2)	6 (75.0)
Diplopia	10 (25.6)	7 (22.6)	3 (37.5)
Facial muscles	2 (5.1)	2 (6.5)	0
Bulbar muscles	9 (23.1)	9 (29.0)	0
Respiratory muscles	16 (41.0)	16 (51.6)	0
Upper limb muscles	9 (23.1)	9 (29.0)	0
Lower limb muscles	12 (30.8)	12 (38.7)	0
COVID-19 severity			
Home management	33 (84.6)	25 (80.6)	8 (100.0)
Hospitalization	6 (15.4)	6 (19.4)	0
Treatment adjustment	12 (30.8)	10 (32.3)	2 (25.0)
Medication escalation	3 (7.7)	3 (9.7)	0
Medication addition	9 (23.1)	7 (22.6)	2 (25.0)
Prognosis			
MMS	6 (15.4)	5 (16.1)	1 (12.5)
Improved	17 (43.6)	14 (45.2)	3 (37.5)
Unchanged	14 (35.9)	10 (32.3)	4 (50.0)
Worse	2 (5.1)	2 (6.5)	0
Improvement (MMS and improved) time, median (range), d	10 (3, 40)	10 (3, 40)	20 (5, 30)

Abbreviations: d, days; MMS, minimal manifestation status.

**Table 5:** The impact of vaccination on infected MG patients.

	Vaccination 1 (*n* = 14)	Vaccination 2 (*n* = 129)	Unvaccination (*n* = 101)	*p*
Gender				0.250
Female	6 (42.9)	51 (39.5)	51 (50.5)	
Male	8 (57.1)	78 (60.5)	50 (49.5)	
Age, median (Q1, Q3), y	62 (50, 73)	58 (44, 68)	58 (42, 68)	0.405
MG type				0.106
gMG	7 (50.0)	67 (51.9)	66 (65.3)	
oMG	7 (50.0)	62 (48.1)	35 (34.7)	
AChR-Ab	11 (78.6)	107 (82.9)	78 (77.2)	0.549
MG duration (Q1, Q3), m	57 (27, 97)	25 (14, 66) *^#^	51 (30, 77)	0.017
Treatment at infection				0.012
No IST	10 (71.4)	75 (58.1)	32 (31.7)	
IST	4 (28.6) ^†^	54 (41.9)	69 (68.3)	
MG symptoms				0.478
MECI	1 (7.1)	24 (18.6)	14 (13.9)	
NMECI	13 (92.9)	105 (81.4)	87 (86.1)	
COVID-19 severity				0.292
Hospitalization	1 (7.1)	8 (6.2)	12 (11.9)	
Home management	13 (92.9)	121 (93.8)	89 (88.1)	

Vaccination 1—patients who received their booster dose within 6 months before COVID-19; Vaccination 2—patients who were vaccinated more than 6 months before COVID-19. * Compared to the vaccination 1 group, *p* < 0.05; # compared to the unvaccination group, *p* < 0.05; † compared to the unvaccination group, *p* < 0.05.

## Data Availability

The datasets generated during and/or analyzed during the current study are available from the corresponding author on reasonable request.

## Supplementary Materials

The following supporting information can be downloaded at: https://www.mdpi.com/article/10.3390/vaccines12111221/s1, Table S1: Clinical characteristics of MG patients receiving COVID-19 inactivated vaccines; Table S2: Course and prognosis of patients with MECV.
