# Supplementary material for: The Impact of COVID-19 Vaccination and Infection on the Exacerbation of Myasthenia Gravis
Source: Vaccines (Basel). 2024 Oct 27;12(11):1221. doi: 10.3390/vaccines12111221 (PMC11598725; doi:10.3390/vaccines12111221)
Supplement: Supplementary file 1 [file vaccines-12-01221-s001.zip › vaccines-3251234-supplementary.pdf]

Table S1. Clinical characteristics of MG patients receiving COVID-19 inactivated vaccines.

	**Total (n=263)**	**MECV (n=7)**	**NMECV (n=256)**	***P***
Gender				
Female	110 (41.8)	2 (28.6)	108 (42.2)	0.740
Male	153 (58.2)	5 (71.4)	148 (57.8)	
Age, median (Q1, Q3), y	64 (56, 71)	56 (47, 71)	56 (41, 64)	0.457
MG Type				
General MG	102 (38.8)	3 (42.9)	99 (38.7)	1.000
Ocular MG				
AChR-Ab	197 (74.9)	6 (85.7)	191 (74.6)	0.821
Thymoma	21 (8.0)	1 (14.3)	20 (7.8)	1.000
MG duration, median (Q1, Q3), m	37 (26, 57)	44 (37, 74)	37 (26, 57)	0.583
MG-ADL scale at vaccination				
0 (stable)	205 (77.9)	6 (85.7)	199 (77.7)	0.655
1-2 (mild)	29 (11.0)	0	29 (11.3)	
3-5 (moderate)	24 (9.1)	1 (14.3)	23 (9.0)	
≥6 (severe)	5 (1.9)	0	5 (2.0)	
MG-ADL score, median (range)	0 (0-9)	0 (0-3)	0 (0-9)	0.654
MG stabilization time before vaccination	12 (5, 24)	36 (4, 48)	12 (5, 23)	0.280
Treatment at vaccination				
No medication	103 (39.2)	3 (42.9)	100 (39.1)	0.764
Pyridostigmine alone	53 (20.2)	2 (28.6)	51 (19.9)	
IST*	107 (40.7)	2 (28.6)	105 (41.0)	
Vaccine type				
Inactivated vaccine	263 (100.0)	7 (100.0)	256 (100.0)	NA
Recombinant protein vaccine	0	0	0	
Adenovirus vector vaccine	0	0	0	
mRNA vaccine	0	0	0	
Immune status				
Finished	247 (93.9)	5 (71.4)	242 (94.5)	0.061
Unfinished	16 (6.1)	2 (28.6)	14 (5.5)	

Abbreviations: MG, myasthenia gravis; MECV, MG exacerbation after COVID-19 vaccination; NMECV: no MG exacerbation after COVID-19 vaccination; y, years; oMG, ocular MG; gMG, general MG; AChR-Ab, acetylcholine receptor antibody; Q1, the first quartile; Q3, the third quartile; m, months; MG-ADL, myasthenia gravis activity of daily living; IST, immunosuppressants.

* IST: including steroids and non-steroids immunosuppressants.

Table S2. Course and prognosis of patients with MECV.

Case No. /Sex/Age, y	Type of vaccines	Time from vaccination to exacerbation	Symptoms		MG-ADL		Treatment		Last Follow-up		
			Baseline	Exacerbation	Baseline	Exacerbation	Baseline	Exacerbation (change in medication)	Follow-up time, m	PIS/ MG-ADL	Days to MMS
1/M/47	Inactivated (CoronaVac)	2 days after 2nd dose	No	Ptosis	0	2	No	PYR 180 mg/d	2	MMS/0	30
2/F/71	Inactivated (CoronaVac)	2 days after 2nd dose	No	Ptosis	0	2	No	PYR 90 mg/d	2	MMS/0	60
3/M/66	Inactivated (CoronaVac)	30 days after 2nd dose	No	Bulbar weakness, Limb weakness	0	5	PYR 120 mg/d	No change (refused IST)	3	Unchanged/5	-
4/M/39	Inactivated (CoronaVac)	14 days after 2nd dose	No	Diplopia	0	3	No	PYR 180 mg/d; GC 20 mg/d	4	MMS/0	15
5/M/81	Inactivated (CoronaVac)	2 days after 1st dose	No	Ptosis	0	3	PYR 90 mg/d	TCM	4	MMS/0	30
6/F/29	Recombinant protein (ZF2001)	16 days after 3rd dose	No	Ptosis, Bulbar weakness, Limb weakness	0	12	No	PYR 360 mg/d; GC 60 mg/d; RTX 600 mg; IVIG	5	Improved/5	-
7/M/50	Inactivated (CoronaVac)	10 days after 1st dose	No	Ptosis, Diplopia	0	5	TAC 0.5 mg/d; GC 5 mg/d	TAC 2 mg/d;	5	MMS/0	150
8/F/56	Inactivated (BBIBP-CorV)	20 days after 2nd dose	Ptosis	Ptosis, Bulbar weakness, Limb weakness	3	12	PYR 180mg/d; GC 20 mg/d	PYR 240 mg/d; GC 60 mg/d	7	MMS/0	30

Abbreviations: No., number; y, years; m, months; PIS, postintervention status; MG-ADL, myasthenia gravis activity of daily living; M, male; F, female; PYR, pyridostigmine; IST, immunosuppressants; GC, glucocorticoids; TCM, Traditional Chinese Medicine; RTX, rituximab; IVIG, intravenous immunoglobulins; TAC, tacrolimus; MMS, minimal manifestation status.
